# Revising the measurement process in the variational quantum eigensolver: is it possible to reduce the number of separately measured operators?†
†Electronic supplementary information (ESI) available. See DOI: 10.1039/c8sc05592k


**DOI:** 10.1039/c8sc05592k

**Published:** 2019-02-12

**Authors:** Artur F. Izmaylov, Tzu-Ching Yen, Ilya G. Ryabinkin

**Affiliations:** a Department of Physical and Environmental Sciences , University of Toronto Scarborough , Toronto , Ontario M1C 1A4 , Canada; b Chemical Physics Theory Group , Department of Chemistry , University of Toronto , Toronto , Ontario M5S 3H6 , Canada . Email: artur.izmaylov@utoronto.ca; c OTI Lumionics Inc. , 100 College Street 351 , Toronto , Ontario M5G 1L5 , Canada

## Abstract

Current implementations of the Variational Quantum Eigensolver (VQE) technique for solving the electronic structure problem involve splitting the system qubit Hamiltonian into parts whose elements commute within their single qubit subspaces. The number of such parts rapidly grows with the size of the molecule. This increases the computational cost and can increase uncertainty in the measurement of the energy expectation value because elements from different parts need to be measured independently. To address this problem we introduce a more efficient partitioning of the qubit Hamiltonian using fewer parts that need to be measured separately. The new partitioning scheme is based on two ideas: (1) grouping terms into parts whose eigenstates have a single-qubit product structure, and (2) devising multi-qubit unitary transformations for the Hamiltonian or its parts to produce less entangled operators. The first condition allows the new parts to be measured in the number of involved qubit consequential one-particle measurements. Advantages of the new partitioning scheme resulting in severalfold reduction of separately measured terms are illustrated with regard to the H_2_ and LiH problems.

## Abstract 2

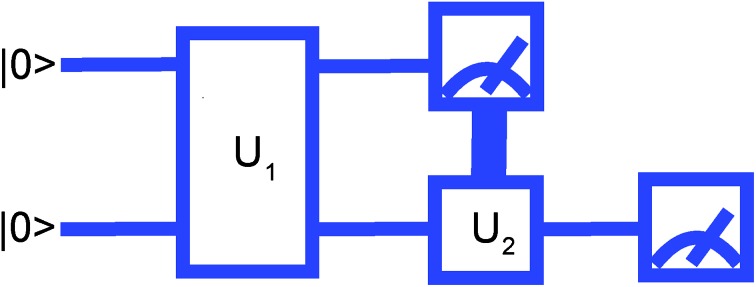
We have introduced two approaches to reduce the number of separately measured terms in molecular Hamiltonians within the Variational Quantum Eigensolver (VQE) technique for solving the electronic structure problem.

## 1 Introduction

One of the most practical schemes for solving the electronic structure problem of current and near-future universal quantum computers is the variational quantum eigensolver (VQE) method.^1^–^5^ This approach involves the following steps: (1) reformulating the electronic Hamiltonian (*Ĥ*_e_) in the second quantized form, (2) transforming *Ĥ*_e_ to the qubit form (*Ĥ*_q_) by applying iso-spectral fermion-spin transformations such as Jordan–Wigner (JW)^6^,^7^ or more resource-efficient Bravyi–Kitaev (BK),^8^–^12^ (3) solving the eigenvalue problem for *Ĥ*_q_ by variational optimization of unitary transformations for a qubit wavefunction. The last step uses a hybrid quantum-classical technique where a classical computer suggests a trial unitary transformation *U*, and its quantum counterpart provides an energy expectation value of *E*_*U*_ = = 〈*Ψ*_0_|*U*^†^*Ĥ*_q_*U*|*Ψ*_0_〉, here |, here |*Ψ*_0_〉 is an initial qubit wavefunction (it is frequently taken as an uncorrelated product of all spin-up states of individual qubits). The two steps, on classical and quantum computers, are iterated till convergence. The VQE was successfully implemented on several quantum computers and used for few small molecules up to BeH is an initial qubit wavefunction (it is frequently taken as an uncorrelated product of all spin-up states of individual qubits). The two steps, on classical and quantum computers, are iterated till convergence. The VQE was successfully implemented on several quantum computers and used for few small molecules up to BeH_2_.^13^

One of the big problems of the VQE is that to calculate *E*_*U*_, the quantum computer measures parts of *H*_q_ rather than the whole *H*_q_ on the *U*|*Ψ*_0_〉 wavefunction. This stems from technological restrictions of what can be currently measured on available architectures. Dramatic consequences of this restriction can be easily understood with the following simple example. Let us assume that wavefunction. This stems from technological restrictions of what can be currently measured on available architectures. Dramatic consequences of this restriction can be easily understood with the following simple example. Let us assume that *Ĥ*_q_ = *Â* + *B[combining circumflex]*, where *Â* and *B[combining circumflex]* are measurable components of *Ĥ*_q_ and [*Â*, *B[combining circumflex]*] ≠ 0, otherwise they could be measured at the same time at least in principle. The actual hardware restrictions on measurable components are somewhat different and will be discussed later, for this illustration these differences are not important. Even if one has an exact eigenstate of *Ĥ*_q_, *U*|*Ψ*_0_〉, measuring it on , measuring it on *Â* or *B[combining circumflex]* would not give a certain result because *Â* and *B[combining circumflex]* do not commute with *Ĥ*_q_. Thus, one would not be able to distinguish the exact eigenstate from other states by its zero variance. The origin of the discrepancy between quantum uncertainty given by the variance (Var) of *Ĥ*_q_ (true uncertainty) and by the sum of variances for *Â* and *B[combining circumflex]* is neglect of covariances (Cov)

1 Var(*Ĥ*_q_) = Var(*Â*) + Var(*B[combining circumflex]*) + Cov(*Â*, *B[combining circumflex]*) + Cov(*B[combining circumflex]*, *Â*),

2 Var(*Â*) = ) = 〈*Â*^2^〉 − 〈 – 〉 − 〈*Â*〉^2^,

3 Cov(*Â*, *B[combining circumflex]*) = ) = 〈*ÂB[combining circumflex]*〉 − 〈 – 〉 − 〈*Â*〉〈〉〈*B[combining circumflex]*〉..

Thus, even though the *Ĥ*_q_ average is equal to averages of *Â* and *B[combining circumflex]*, the true quantum uncertainty of *Ĥ*_q_ is overestimated by a sum of variances for *Â* and *B[combining circumflex]*. Moreover, the number of measurements to sample *Â* and *B[combining circumflex]* is twice as many as that for *Ĥ*_q_ if the eigenstate nature of *U*|*Ψ*_0_〉 is not known is not known *a priori*.

The variance of any Hamiltonian depends only on the Hamiltonian and the wavefunction, but if one approximates the variance using only variances of Hamiltonian parts and neglects covariances between the parts, the result of such an approximation will depend on the partitioning. Importantly, the sum of variances for the Hamiltonian parts can either under- or overestimate the true Hamiltonian variance. To see how ignoring covariances can erroneously make estimates of the uncertainty arbitrarily small consider an artificial example, where the Hamiltonian variance is measured as *n* independent measurements of its *Ĥ*_q_/*n* identical parts. Due to the linear scaling of the variance sum with *n* and the inverse quadratic scaling of variances of individual terms with *n*, the overall scaling of the variance is inversely proportional to *n* and can be made arbitrarily small by choosing large enough *n*. This follows from a wrong assumption that parts (*Ĥ*_q_/*n*) are independent and covariances between them are zero.

Generally, the number of non-commuting terms in *Ĥ*_q_ grows with the size of the original molecular problem, and the total uncertainty from the measurement of individual terms will increase. This increase raises the standard deviation of the total measurement process and leads to a large number of measurements to reach convergence in the energy expectation value. The question we would like to address is whether it is possible to reduce the number of the *Ĥ*_q_ terms that needs to be measured separately.

In this paper we introduce a new systematic approach to decreasing uncertainty of the expectation energy measurement. We substitute the conventional measurement partitioning of the Hamiltonian with groups of qubit-wise commuting operators^13^,^14^ by partitioning to terms whose eigenstates can be found exactly using the mean-field procedure. Owing to a more general structure of such terms the Hamiltonian can be split into a fewer number of them. Interestingly, the general operator conditions on such mean-field terms have not been found in the literature and have been derived in this work for the first time. To decrease the number of these terms even further, we augment the mean-field treatment with few-qubit unitary transformations that allow us to measure few-qubit entangled terms. Measurement of newly introduced terms requires the scheme appearing in the cluster-state quantum computing,^15^,^16^ it is qubit-wise measurement with use of previous measurement results to define what single-qubit operators to measure next.

## 2 Theory

### 2.1 Qubit Hamiltonian

In order to formulate the electronic structure problem for a quantum computer that operates with qubits (two-level systems), the electronic Hamiltonian needs to be transformed iso-spectrally to its qubit form. This is done in two steps. First, the second quantized form of *Ĥ*_e_ is obtained4
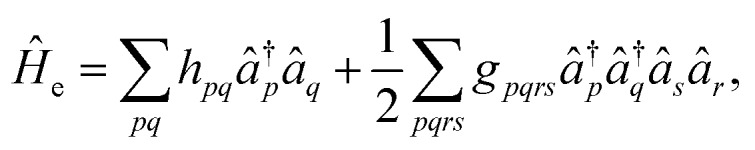
where *â*†*p* (*â*_*p*_) are fermionic creation (annihilation) operators, *h*_*pq*_ and *g*_*pqrs*_ are one- and two-electron integrals in a spin-orbital basis.^17^ This step has polynomial complexity and is carried out on a classical computer. Then, using the JW^6^,^7^ or more resource-efficient BK transformation,^8^–^12^ the electronic Hamiltonian is converted iso-spectrally to a qubit form5
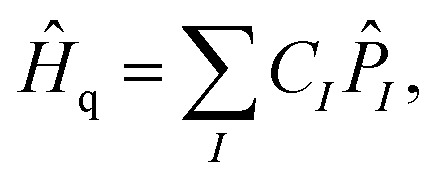
where *C*_*I*_ are numerical coefficients, and *P[combining circumflex]*_*I*_ are Pauli “words”, products of Pauli operators of different qubits

6 *P[combining circumflex]*_*I*_ = ···*σ̂*_2_^(*I*)^*σ̂*_1_^(*I*)^,

*σ̂*_*i*_^(*I*)^ is one of the *x[combining circumflex]*, *ŷ*, *ẑ* Pauli operators for the *i*^th^ qubit. The number of qubits *N* is equal to the number of spin-orbitals used in the second quantized form [eqn (4)]. Since every fermionic operator is substituted by a product of Pauli operators in both JW and BK transformations, the total number of Pauli words in *Ĥ*_q_ scales as *N*^4^.

### 2.2 Conventional measurement

In the conventional VQE scheme the *Ĥ*_q_ is separated into sums of qubit-wise commuting (QWC) terms,7
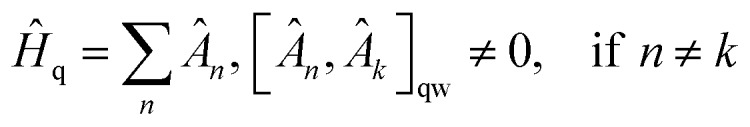

8
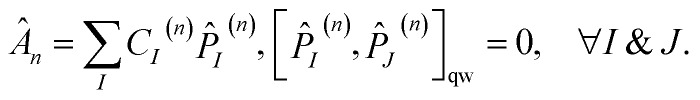
Here [*P[combining circumflex]*_*I*_^(*n*)^, *P[combining circumflex]*_*J*_^(*n*)^]_qw_ denotes a qubit-wise commutator of two Pauli words, it is zero only if all one-qubit operators in *P[combining circumflex]*_*I*_^(*n*)^ commute with their counterparts in *P[combining circumflex]*_*J*_^(*n*)^. Clearly, if [*P[combining circumflex]*_*I*_^(*n*)^, *P[combining circumflex]*_*J*_^(*n*)^]_qw_ then the normal commutator is [*P[combining circumflex]*_*I*_^(*n*)^, *P[combining circumflex]*_*J*_^(*n*)^] = 0. The opposite is not true, a simple example is [*x[combining circumflex]*_1_*x[combining circumflex]*_2_, *ŷ*_1_*ŷ*_2_] = 0 but [*x[combining circumflex]*_1_*x[combining circumflex]*_2_, *ŷ*_1_*ŷ*_2_]_qw_ ≠ 0. We will not be using non-zero results of the qubit-wise commutator and therefore their exact values are not important, but it is assumed that [.,.]_qw_ is bi-linear for both operators.

Partitioning of the *H*_q_ in eqn (7) allows one to measure all Pauli words within each *Â*_*n*_ term in a single set of *N* one-qubit measurements. For every qubit, it is known from the form of *Â*_*n*_, what Pauli operator needs to be measured. The advantage of this scheme is that it requires only single-qubit measurements, which are technically easier than multi-qubit measurements. The disadvantage of this scheme is that the Hamiltonian may require measuring too many *Â*_*n*_ terms separately.

A natural extension of partitioning in eqn (7) is to sum more general terms9
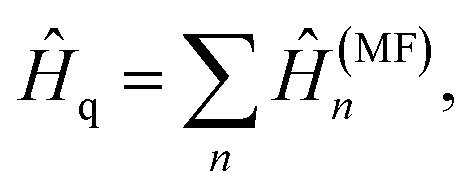
with the condition that *Ĥ*(MF)*n* eigenstates can be presented in a single-product form of single-qubit wavefunctions. In other words, the eigenstates of the *Ĥ*(MF)*n* fragments are unentangled and can be obtained using a mean-field procedure. This condition would allow measurement of each *Ĥ*(MF)*n* fragment qubit after a qubit. However, to perform the new splitting we need an exact definition of the mean-field (MF) Hamiltonian so that we can recognize these new blocks within the total Hamiltonian.

### 2.3 Mean-field Hamiltonians

What is the most general form of a qubit Hamiltonian whose eigenstates can be presented as single factorized products of one-qubit wavefunctions? Note that the well-known example of such Hamiltonians, separable operators,10
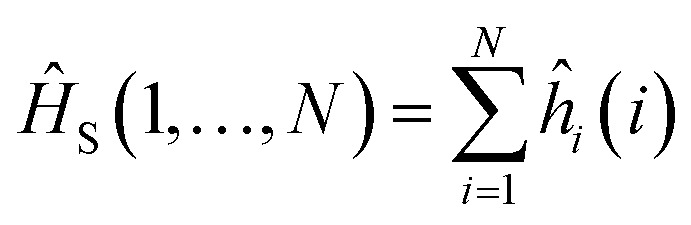
are a particular class that does not provide the most general form. In other words, there are many more Hamiltonians that are not separable but are still in the MF class, one simple example is

11 *Ĥ*_MF_(1,2) = *x[combining circumflex]*_2_ + *ẑ*_1_*ŷ*_2_,

which does not follow the form of eqn (10) but whose eigenstates, |+_*z*_〉_1_|±_*x*+*y*_〉_2_ and |–*z*〉_1_|±_*x*–*y*_〉_2_,‡
‡Here, we use the notation |±_*σ*_〉_*n*_ for the *n*^th^ qubit eigenstates of a σ one-particle operator with ±1 eigenvalues. are unentangled products.

We formulate the general criterion for a Hamiltonian *H*(1,…*N*) to be in the MF class as follows. There should exist *N* one-particle operators {*Ô*_*k*_(*k*)}*Nk* = 1§
§To simplify the notation we use freedom in qubit enumeration and assume that we work with the qubit enumeration that follows the described reductive sequence. that commute [*Ô*_*k*_, *Ĥ*_*N*–*k*+1_] = 0 with the system of *N* Hamiltonians {*Ĥ*_*N*–*k*+1_}*Nk* = 1 constructed in the following way that we will refer as a reductive chain:12
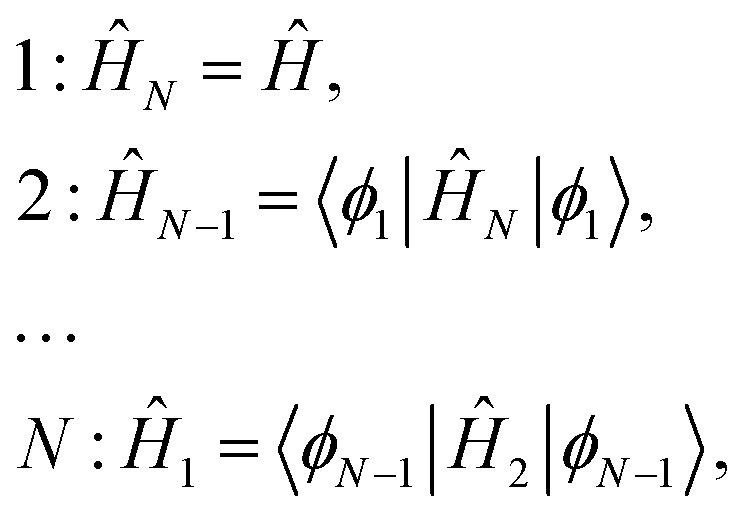
where *Ô*_*k*_|*φ*_*k*_〉 = = *λ*_*k*_|*φ*_*k*_〉. The final operator in this chain is a one-particle operator that commutes with itself and defines . The final operator in this chain is a one-particle operator that commutes with itself and defines *Ô*_*N*_ = *Ĥ*_1_. The proof of this criterion can be found in Appendix A. It is easy to see that 
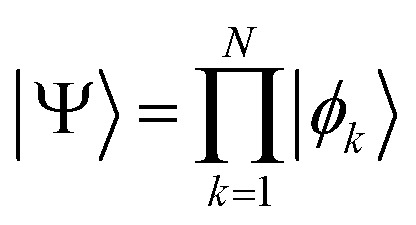
 is an eigenfunction of *Ĥ*. Clearly, separable Hamiltonians are in the MF class because for them, *Ô*_*k*_'s can be taken as *ĥ*_*k*_(*k*) from eqn (10). However, note that because the system of *Ô*_*k*_ operators is required to commute not with *Ĥ* but with the reduced set of Hamiltonians, the formulated criterion goes beyond separable Hamiltonians.

A general procedure to determine whether a particular qubit Hamiltonian *Ĥ* is in the MF class or not requires finding all *N* one-particle operators *Ô*_*k*_. The procedure starts with a check whether there is at least one qubit *k* for which

13 [*Ĥ*, (*ax[combining circumflex]*_*k*_ + *bŷ*_*k*_ + *ccẑ*_*k*_)] = 0

can be achieved by choosing a non-zero vector (*a*, *b*, *c*). Once the first operator *Ô*_1_(*k*) = *ax[combining circumflex]*_*k*_ + *bŷ*_*k*_ + *ccẑ*_*k*_ is found its eigenstates can be integrated out to generate *Ĥ*_*N*–1_, and the procedure can be repeated to find *Ô*_2_ that commutes with *Ĥ*_*N*–1_.

### 2.4 Measurement of mean-field Hamiltonians

Measuring an *N*-qubit mean-field Hamiltonian can be done by performing a single set of sequential *N* one-qubit measurements. Each qubit projective measurement in this set will collapse the measured wavefunction to an eigenstate of the corresponding single qubit operator. The single qubit operators that need to be measured are *Ô*_*k*_'s operators. The definition of one particle operators may depend on the result of the previous measurement. Let us consider the mean-field Hamiltonian in eqn (11): *Ô*_1_(1) = *ẑ*_1_, and *Ô*_2_(2) = *x[combining circumflex]*_2_ ± *ŷ*_2_, where ± is determined by the eigenfunction chosen from the *Ô*_1_ spectrum to generate the *Ĥ*_1_ = = 〈*φ*_1_^±^|*Ĥ*_MF_|*φ*_1_^±^〉 in the chain of in the chain of eqn (12). This ambiguity does not allow one to present *Ĥ*_MF_ as an operator with all qubit-wise commuting components. An attempt on this can be done by inserting the projectors on the eigenstates of *ẑ*_1_ instead of the operator:

14 *Ĥ*_MF_ = (*x[combining circumflex]*_2_ + *ŷ*_2_)|*φ*_1_^+^〉〈〉〈*φ*_1_^+^| + (*x[combining circumflex]*_2_ – *ŷ*_2_)|*φ*_1_^–^〉〈〉〈*φ*_1_^–^|

15 *Ĥ*_MF_ = [(*x[combining circumflex]*_2_ + *ŷ*_2_)(1 + *ẑ*_1_) + (*x[combining circumflex]*_2_ – *ŷ*_2_)(1 – *ẑ*_1_)]/2,

where *ẑ*_1_|*φ*_1_^±^〉 = ±| = ±|*φ*_1_^±^〉, and even though the projectors onto the |, and even though the projectors onto the |*φ*_1_^±^〉 eigenstates commute, the ( eigenstates commute, the (*x[combining circumflex]*_2_ ± *ŷ*_2_) parts do not.

**Fig. 1:**
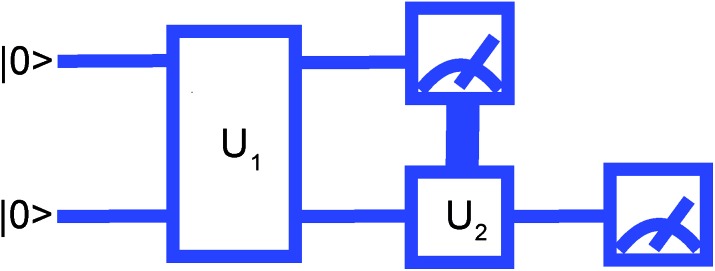
Measurement where the second qubit is rotated by *U*_2_ depending on the result of the first qubit measurement.

Therefore, the scheme for measuring the *Ĥ*_MF_ will be as shown in Fig. 1. Note that no matter how entangled the initial wavefunction is, measuring *Ĥ*_MF_ does not require measuring *x[combining circumflex]*_2_ and *ẑ*_1_*ŷ*_2_ separately as was done in the regular VQE scheme.

In practice, qubit-wise measurements using previous measurement results to define what single-qubit operators to measure next, or feedforward measurements, have been implemented in quantum computers based on superconductor and photonic qubit architectures.^18^,^19^ The essential feasibility condition for the feedforward measurement is that the delay introduced by measurements is much shorter than the qubit coherence time. For superconducting (photonics) qubit architectures this condition has been achieved with typical timescales for a measurement and coherence as 2 μs (ref. 20) (150 ns (ref. 19)) and 40 μs (ref. 21) (100 ms (ref. 22)), respectively.

### 2.5 Mean-field partitioning

Even though regular molecular qubit Hamiltonians are not guaranteed to be in the MF class, it is always possible to split any *N*-qubit Hamiltonian into a sum of MF Hamiltonians. To see this, we will present a heuristic partitioning scheme that guarantees the MF partitioning.

Our scheme uses ranking of all qubits *k* = 1,…,*N* based on a geometrical characteristic *l*(*k*), which is defined as follows. For an arbitrary qubit *k*, the total Hamiltonian can be written as

16 *Ĥ* = *ĥ*_*x*_*x[combining circumflex]*_*k*_ + *ĥ*_*y*_*ŷ*_*k*_ + *ĥ*_*z*_*ẑ*_*k*_ + *ĥ*_*e*_

where *ĥ*_*x*,*y*,*z*,*e*_ are the residual operators that do not contain Pauli matrices for the *k*^th^ qubit. Assembling coefficients of Pauli words in operators *ĥ*_*x*,*y*,*z*_ into vectors, *h[combining macron]*_*x*,*y*,*z*_, we build matrix *A*_*k*_ = [*h[combining macron]*_*x*_*h[combining macron]*_*y*_*h[combining macron]*_*z*_] with dimensions *M* by 3, where *M* is the number of different Pauli words in *ĥ*_*x*,*y*,*z*_ operators. To define *l*(*k*), we evaluate matrix *S*_*k*_ = *A*†*kA*_*k*_ and assign *l*(*k*) = dim(ker(*S*_*k*_)). Evaluating *S*_*k*_ is equivalent to obtaining the overlap between three vectors *h[combining macron]*_*x*,*y*,*z*_ assuming the orthogonal basis, while the dimensionality of its kernel is the number of its zero eigenvalues.

*l*(*k*) allows one to answer a question on whether there is a transformation involving only the *k*^th^ qubit that can present *Ĥ* in one of the two forms:

17 *Ĥ* = *ĥÔ*_*k*_ + *ĥ*_*e*_,

18
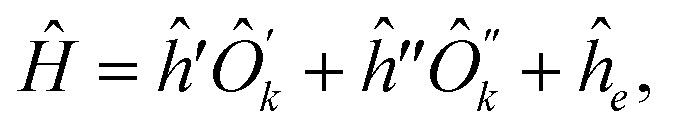
where 
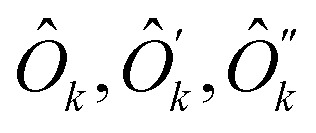
 are operators containing only the *k*^th^ qubit, and *ĥ*, *ĥ*′, *ĥ*′′ are the complementary operators that exclude the *k*^th^ qubit. The positive answers in the forms of eqn (17) and (18) correspond to *l*(*k*) = 2 and *l*(*k*) = 1, respectively. *l*(*k*) = 2 is equivalent to the MF condition of eqn (13), with *Ô*_*k*_ = *ax[combining circumflex]*_*k*_ + *bŷ*_*k*_ + *ccẑ*_*k*_. For *l*(*k*) = 1, the MF treatment of the *k*^th^ qubit is not possible but using eqn (18) the *k*^th^ qubit dependence in the Hamiltonian can be somewhat compactified. Coefficients for 
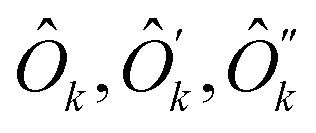
 and *ĥ*, *ĥ*′, *ĥ*′′ operators can be found from non-zero eigenvectors of *S*_*k*_ (this process is detailed in Appendix B). The negative answer to the question leaves *Ĥ* in the original form of eqn (16) and is equivalent to *l*(*k*) = 0.

**Fig. 2:**
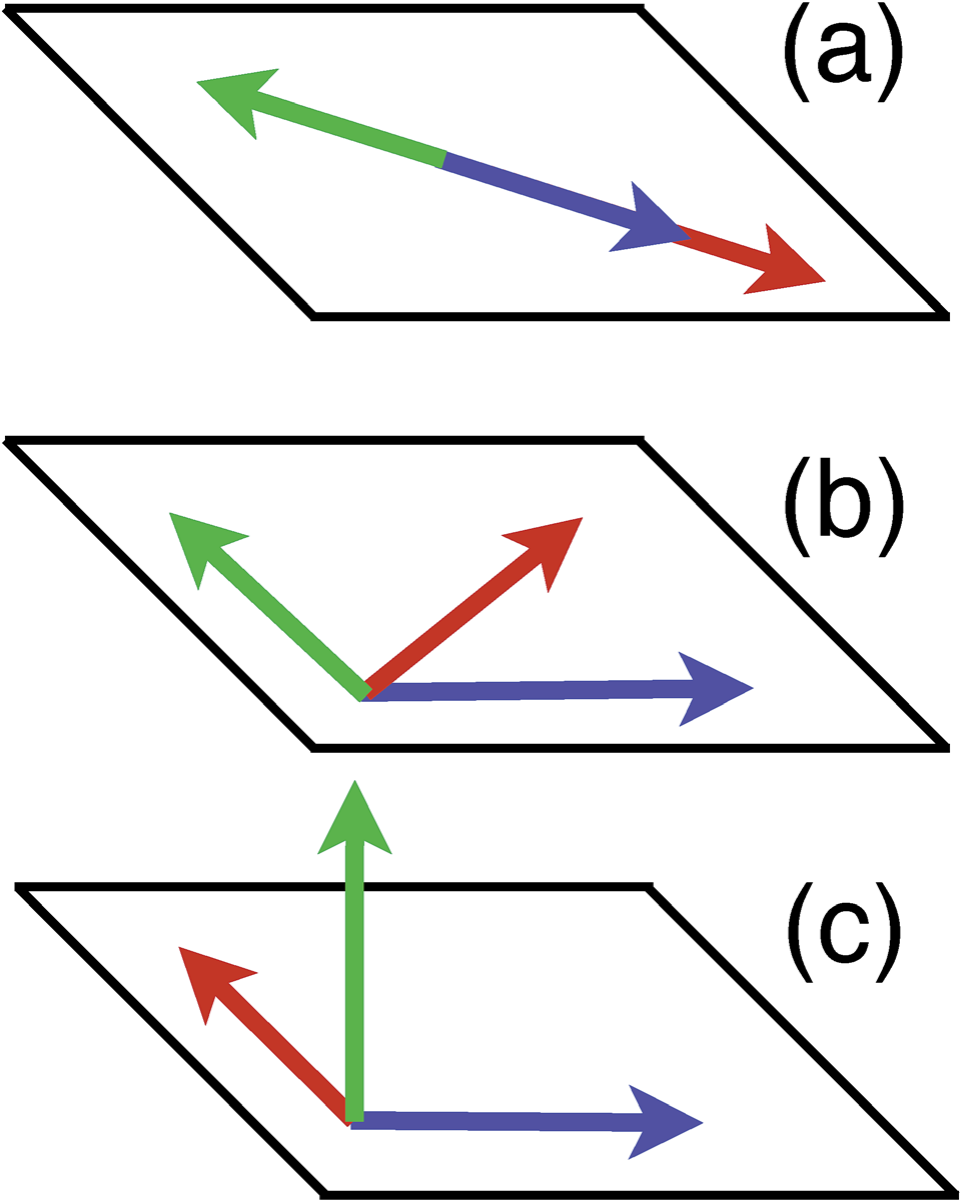
Possible geometrical arrangement of three multi-dimensional vectors *h[combining macron]*_*x*,*y*,*z*_ (green, blue, and red arrows): (a) collinear arrangement [*l*(*k*) = 2], (b) planar arrangement [*l*(*k*) = 1], (c) linearly independent case [*l*(*k*) = 0].

The question about possible compactification of the *k*^th^ qubit dependence in the Hamiltonian has a simple geometric interpretation in terms of arrangement of the three vectors *h[combining macron]*_*x*,*y*,*z*_. These multi-dimensional vectors can be linearly independent (eqn (16)), located within some plane (eqn (18)), or collinear to each other (eqn (17)), Fig. 2 illustrates all three cases.

**Fig. 3:**
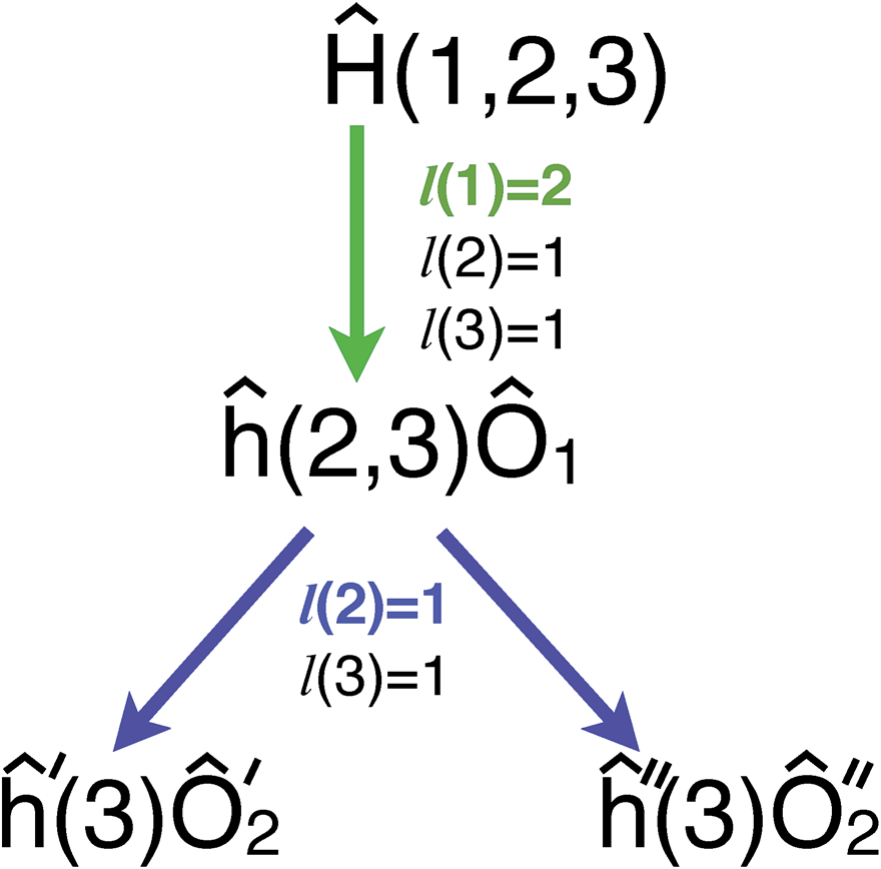
The MF partitioning scheme uses the *l*(*k*) function at each step to split a three qubit Hamiltonian (detailed in Appendix B) into two fragments. The MF partitioned form is 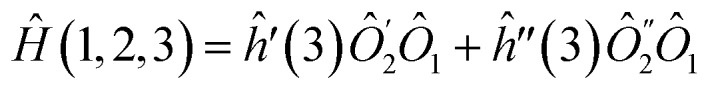, where all qubits in both fragments can be treated using the MF procedure.

Using a set of *l*(*k*)'s for a given Hamiltonian one can decide how many qubits can be treated using the MF procedure, these will be all qubits with *l*(*k*) = 2. Once all of such qubits have been considered, the MF partitioning of *l*(*k*) = 1 qubits begins. For *l*(*k*) = 1, the Hamiltonian can be split for any of such qubits into two parts: 
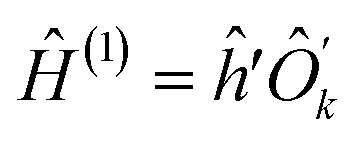
 and 
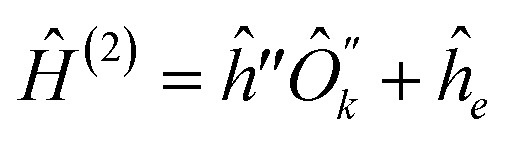
. In both parts the *k*^th^ qubit can be treated using the MF treatment, which allows one to continue the consideration for *ĥ*′, *ĥ*′′ and *ĥ*_*e*_. Finally, if only qubits with *l*(*k*) = 0 are left, then *Ĥ* needs to be partitioned to three Hamiltonians *Ĥ*^(1)^ = *ĥ*_*x*_*x[combining circumflex]*_*k*_, *Ĥ*^(2)^ = *ĥ*_*y*_*ŷ*_*k*_, and *Ĥ*^(3)^ = *ĥ*_*z*_*ẑ*_*k*_ + *ĥ*_*e*_, where at least the *k*^th^ qubit can be treated using MF. After this separation one can apply the reduction chain to each of the three operators. Fig. 3 illustrates the partitioning for a three qubit case detailed in Appendix B. In the case when reducing the *k*^th^ qubit does not produce a Hamiltonian with reducible qubits the partitioning needs to be repeated, as in Fig. 3 when transforming qubit 1 led to *h*(2,3) where none of the qubits can be reduced.

Our scheme can be considered as an example of a greedy algorithm because at every step it tries to find locally the most optimal reduction, a qubit with the highest *l*(*k*). The reduction is only possible if there is linear dependency between complementary vectors 
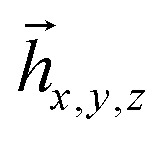
. The lower the dimensionality of the linear space, where these vectors are located, the more probable such linear dependence. Thus, treating qubits with the highest *l*(*k*) first is justified by the reduction of the space dimensionality along the reductive scheme. In the example of Fig. 3 treatment of qubits 2 and 3 in the beginning would require partitioning of the Hamiltonian to two branches for each of them, while leaving the 3^rd^ qubit to the end did not generate any new terms for it.

It is possible that more than one qubit will have the highest *l*(*k*). To do more optimal selection in this case, one would need to consider maxima of *l*(*k*) functions on qubits that enter complementary Hamiltonians *ĥ* for different reduction candidates. This consideration makes the partitioning computationally costly and was not performed in this work.

Applying the partitioning scheme guarantees to result in a sum of MF Hamiltonians that can be measured in *N*-qubit one-particle measurements. Since any linear combination of QWC terms form a MF Hamiltonian, this partitioning scheme cannot produce more terms than those used in the regular VQE measuring scheme.

### 2.6 Unitary transformations generating mean-fields

Partitioning the non-MF blocks in the Hamiltonian to obtain more MF terms leads to growth of the terms needed to be measured. An alternative treatment of non-MF groups is to search for multi-qubit operators that commute with them. Finding such operators may lead to unitary transformations that can transform non-MF Hamiltonians into Hamiltonians where qubits shared with the commuting operator can be treated using the mean-field procedure. Similar search for multi-qubit operators commuting with the system Hamiltonian was used recently by Bravyi and coworkers to reduce the qubit count in the conventional VQE scheme.^23^

Let us consider an example where an *N*-qubit non-MF Hamiltonian *Ĥ* has a two-qubit operator *Ô*^(2)^(1,2) commuting with it (without loss of generality we can assume that *Ô*^(2)^ acts on the first two qubits). Then, under certain conditions detailed in Appendix A, *Ĥ* allows for its eigenstates Ψ to be written as *Ψ*(1,…*N*) = *Φ*(1,2)*ψ*(3,…*N*), where *Φ*(1,2) is an eigenstate of *Ô*^(2)^. One can always write *Φ*(1,2) = *Û*(1,2)*φ*_1_(1)*φ*_2_(2), where *Û*(1,2) is an operator entangling the product state *φ*_1_(1)*φ*_2_(2) into *Φ*(1,2). Using this unitary operator, one can obtain the Hamiltonian *Ĥ*_12_ = *Û*(1,2)^†^*ĤÛ*(1,2) that has an eigenstate *Ψ*_12_(1,…*N*) = *φ*_1_(1)*φ*_2_(2)*ψ*(3,…*N*) where qubits 1 and 2 are unentangled. Therefore, there should be one-particle operators of qubits 1 and 2 that commute with *Ĥ*_12_ and its MF-reduced counterpart. Finding these operators and their eigenfunctions *φ*_1_(1) and *φ*_2_(2) allows us to integrate out qubits 1 and 2

19 *Ĥ*_*N*–2_ = = 〈*φ*_1_*φ*_2_|*H*_12_|*φ*_1_*φ*_2_〉..

Search for one- or multi-qubit operators commuting with *Ĥ*_*N*–2_ can be continued. The procedure to find commuting operators with increasing number of qubits requires exponentially increasing number of variables parametrizing such operators. Indeed, a *k*-qubit operator requires a 3^*k*^ coefficient for all Pauli words in commutation equations similar to eqn (13), also the number of different *k*-qubit operators among *N* qubits is *C*_*N*_^*k*^ ∼ *N*_*k*_. Potentially, such operators always exist (*e.g.*, projectors on eigenstates of the Hamiltonian) but the amount of resources needed for their search can exceed what is available. Thus we recommend interchanging this search with the partitioning described above if the multi-qubit search requires going beyond 2-qubit operators.

To illustrate the complete scheme involving multi-qubit transformations, let us assume that we can continue the reduction chain for *Ĥ* = *Ĥ*_*N*_ by generating the set of Hamiltonians {*Ĥ*_*N*_, *Ĥ*_*N*–2_,…,*Ĥ*_*k*_} using qubit unitary transformations {*U*(1,2), *U*(3,4,5),…,*U*(*N* – *k*,…*N*)} and integrating out variables from *N* to *k*. To take advantage of this reduction chain in measuring an expectation value of an arbitrary wavefunction *χ*(1,…*N*) on *Ĥ*, such a measurement should be substituted by the following set of conditional measurements:

Step 1: first two qubits are measured using *Ĥ*_12_ and the unitary transformed function |*Û*(1,2)^†^*χ*〉 because because20
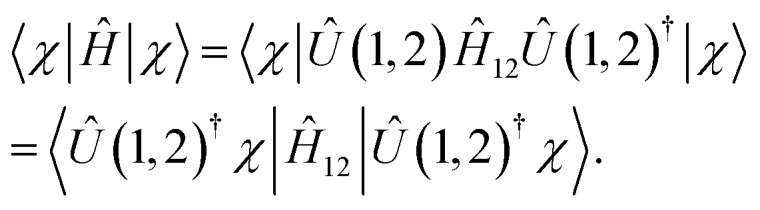



Depending on the results of these measurements the operator *Ĥ*_*N*–2_ is formulated and its unitary transformation *U*(3,4,5) is found. *U*(3,4,5) gives rise to the transformed Hamiltonian *Ĥ*_35_ = *Û*(3,4,5)^†^*Ĥ*_*N*–2_*Û*(3,4,5). The wavefunction after measuring qubits 1 and 2 is denoted as |*χ*_12_〉..

Step 2: qubits 3–5 are measured on *Ĥ*_35_ sequentially using the transformed wavefunction *Û*(3,4,5)^†^|*χ*_12_〉. Results of these measurements will define the next reduction step and the wavefunction that should be unitarily transformed for the next measurement.. Results of these measurements will define the next reduction step and the wavefunction that should be unitarily transformed for the next measurement.

These steps can be continued until all qubits have been measured. If resources allow for finding corresponding multi-qubit unitary transformations, the *Ĥ* Hamiltonian can be measured in *N* single-qubit measurements.

## 3 Numerical studies and discussion

To assess our developments we apply them to the Hamiltonians of the H_2_ and LiH molecules obtained within the STO-3G basis and used to illustrate the performance of quantum computing techniques previously.^13^,^24^,^25^

### 3.1 H_2_ molecule

The BK-transformed qubit Hamiltonian contains the following terms:

21 *Ĥ*_H_2__ = *C*_0_ + *C*_1_*ẑ*_2_ + *C*_2_*ẑ*_3_ + *C*_3_*ẑ*_4_ + *C*_4_*ẑ*_1_*ẑ*_3_ + *C*_5_*ẑ*_2_*ẑ*_4_ + *C*_6_*ẑ*_3_*ẑ*_4_ + *C*_7_*ẑ*_1_*ẑ*_2_*ẑ*_3_ + *C*_8_(1 + *ẑ*_1_)*ẑ*_2_*ẑ*_3_*ẑ*_4_ + *C*_9_*ẑ*_1_*ẑ*_2_*ẑ*_4_ + *C*_10_(1 + *ẑ*_1_)*ŷ*_2_*ẑ*_3_*ŷ*_4_ + *C*_11_(1 + *ẑ*_1_)*x[combining circumflex]*_2_*ẑ*_3_*x[combining circumflex]*_4_.

where some of the *C*_*i*_'s are equal, but it is not going to be important for us (the details of generating this Hamiltonian are given in Appendix C). Clearly *Ĥ*_H_2__ contains three groups of QWC terms, the first three lines form one group, and the two last terms fall into two other groups. *Ĥ*_H_2__ is not a MF Hamiltonian, only qubits 1 and 3 have one-particle operators commuting with the Hamiltonian, while after their reduction the reduced Hamiltonian does not commute with any one-particle operator

22 *Ĥ*_24_ = *D*_0_ + *D*_1_*ẑ*_2_ + *D*_2_*ẑ*_4_ + *D*_3_*ẑ*_2_*ẑ*_4_ + *D*_4_*x[combining circumflex]*_2_*x[combining circumflex]*_4_ + *D*_5_*ŷ*_2_*ŷ*_4_,

where *D*_*i*_'s are constants. Partitioning of *Ĥ*_24_ to three terms using qubit 2 or 4 would not be more efficient than partitioning *Ĥ*_H_2__ in 3 groups of QWC terms from the beginning. However, there is the two-particle operator *ẑ*_2_ẑ_4_ that commutes with *Ĥ*_24_, and it can be used to devise a unitary transformation bringing *Ĥ*_24_ to the MF form. Note that even though *ẑ*_2_*ẑ*_4_ has a spectral degeneracy, this degeneracy will not create problematic entanglement discussed in Appendix A, because there are no other qubits besides 2 and 4 in *Ĥ*_24_. The sought unitary transformation is *U*(2,4) = exp[–i(3π/2)*ẑ*_2_*x[combining circumflex]*_4_], and the transformed MF Hamiltonian is

23 *U*(2,4)^†^*Ĥ*_24_*U*(2,4) = *E*_0_ + *E*_1_*ẑ*_2_ + *E*_2_*ŷ*_2_ + *E*_3_*ŷ*_4_ + *E*_4_*ŷ*_2_*ŷ*_4_ + *E*_5_*ẑ*_2_*ŷ*_4_,

where *E*_*i*_'s are some constants and the first one-particle commuting operator is *Ô*_1_^(4)^ = *ŷ*_4_. After integrating out *Ô*_1_'s eigenfunction, *Ô*_2_^(2)^ is a linear combination of *ẑ*_2_ and *ŷ*_2_.

To illustrate the superiority of the scheme with the use of *U*(2,4) and measurements of the MF Hamiltonian over the regular approach with splitting *Ĥ*_H_2__ to three groups of QWC operators, Table 1 presents variances for the Hamiltonian expectation value for two wavefunctions, the exact eigenfunction (*Ψ*_QCC_) of and the mean-field approximation (*Ψ*_QMF_) to the ground state of the H_2_ problem at *R*(H–H) = 1.5 Å.^25^ The exact solution measured in the new scheme (MF-partitioning 2p) gives only one value with zero variance, while the regular schemes give three distributions for each non-commuting term.

**Table 1:** Estimates of total variances (Var) for the H_2_ and LiH molecules with different partitioning approaches and wavefunctions (*Ψ*_QCC_ from the qubit coupled cluster method,^25^ and *Ψ*_QMF_ from the qubit mean-field approach^26^)^*a*^

Approach	Number of terms	Var (*Ψ*_QCC_)	Var (*Ψ*_QMF_)
**H**_**2**_			
QWC-partitioning	3	0.044	0.026
MF-partitioning 2p	1	0	0.053
〈*Ĥ*_H_2__^2^〉 − 〈 – 〉 − 〈*Ĥ*_H_2__〉^2^	1	0	0.053

**LiH**			
QWC-partitioning	25	0.043	0.037
MF-partitioning 1p	13	0.029	0.036
MF-partitioning 2p	5	0.030	0.038
〈*Ĥ*_LiH_^2^〉 − 〈 – 〉 − 〈*Ĥ*_LiH_〉^2^	1	5.6 × 10^–4^	0.027

^*a*^The number of terms corresponds to the number of separately measured *N*-qubit terms. For all partitionings, covariances have not been included in the Var estimates, which simulates practical estimation of the total variance.

In the approximate wavefunction case, the true variance obtained from the Hamiltonian is larger than that of the conventional approach. This is a consequence of ignoring covariances in the conventional approach. The MF partitioning 2p variance is equal to the exact one, since it is obtained from measuring a single term (the MF Hamiltonian in eqn (23)) and thus does not neglect any covariances.

### 3.2 LiH molecule

We will consider the LiH molecule at *R*(Li–H) = 3.2 Å, it has a 6-qubit Hamiltonian containing 118 Pauli words (see Appendix C for details). This qubit Hamiltonian has 3^rd^ and 6^th^ stationary qubits, which allow one to replace the corresponding *ẑ* operators by their eigenvalues, ±1, thus defining the different “sectors” of the original Hamiltonian. Each of these sectors is characterized by its own 4-qubit effective Hamiltonian. The ground state lies in the *z*_3_ = –1, *z*_6_ = 1 sector; the corresponding 4-qubit effective Hamiltonian (*Ĥ*_LiH_) has 100 Pauli terms. Integrating out 3^rd^ and 6^th^ qubits can be done in the MF framework. The MF treatment of *Ĥ*_LiH_ is not possible without its partitioning.

Before discussing partitioning of *Ĥ*_LiH_ it is worth noting that there are two 2-qubit operators commuting with *Ĥ*^(4)^ (we re-enumerate qubits after the reduction from 6 to 4 qubits in the Hamiltonian)

24 *Ô*_1_^(2)^ = –*ẑ*_1_ + *ẑ*_2_ – *ẑ*_1_*ẑ*_2_

25 *Ô*_2_^(2)^ = –*ẑ*_3_ + *ẑ*_4_ + *ẑ*_3_*ẑ*_4_.

Unfortunately, both operators have degenerate spectra with a single non-degenerate eigenstate and three degenerate states. Moreover, these degeneracies do not satisfy the factorability condition introduced in Appendix A thus proving it impossible to find 2-qubit unitary transformation that would factorize qubits 1 and 2 or 3 and 4.

Table 1 summarizes results of partitioning for *Ĥ*_LiH_ and variances calculated for different wavefunctions and partitioning schemes. The partitioning involving only one-qubit transformations (MF-partitioning 1p) reduces the number of QWC terms by half. Involving the two-qubit transformations at the step before the last one in the MF partitioning reduces the number of terms to only 5 (MF-partitioning 2p), which is a fivefold reduction compared to the conventional QWC form. Alternative pathways in the MP partitioning scheme related to different choices of partitioned qubits with the same value of *l*(*k*) generated not more than 15 and 9 terms for MF partitioning 1p and 2p, respectively. As discussed previously, the qubit mean-field (*Ψ*_QMF_) and qubit coupled cluster (*Ψ*_QCC_) wavefunctions are considered, with the only difference that *Ψ*_QCC_ is a very accurate but not exact ground state wavefunction for LiH (thus there is a small but non-zero variance of the *Ĥ*_LiH_ on *Ψ*_QCC_). Details on the generation of these functions can be found in ref. 25. Variances across different partitionings do not differ appreciably and the main advantage of the MF-partitioning schemes is in the reduction of the number of terms that need to be measured.

## 4 Conclusions

We have introduced and studied a new method for partitioning of the qubit Hamiltonian in the VQE approach to the electronic structure problem. The main idea of our approach is to find Hamiltonian fragments that have eigenstates consisting of single products of one- and two-qubit wavefunctions. The most general criterion for identifying such Hamiltonian fragments was derived for the first time. Once such fragments are found the total wavefunction of the system can be measured on a fragment Hamiltonian in a single pass of *N* single-qubit measurements intertwined with one- and two-qubit rotations that are defined on-the-fly from results of previous qubit measurements. The main gain from such a reformulation is a decrease of separately measured Hamiltonian fragments. Indeed, illustrations on simple molecular systems (H_2_ and LiH) show three- and five-fold reductions of the number of terms that are needed to be measured with respect to the conventional scheme.

In the process of deriving our partitioning procedure, we discovered criteria for eigenstate factorability for an arbitrary Hamiltonian acting on *N* distinguishable particles. Our criteria involve search for few-body operators commuting with the Hamiltonian of interest. Even though the criteria for factorability are exact, realistic molecular Hamiltonians do not satisfy them in general. Therefore, we needed to introduce a heuristic partitioning procedure (greedy algorithm) that splits the system Hamiltonian to fragments that have factorable eigenstates. Even though the procedure does not guarantee the absolutely optimal partitioning to the smallest number of terms, it does not produce more terms than the number of qubit-wise commuting sub-sets.

Interestingly, when one is restricted with single-qubit measurements, the commutation property of two multi-qubit operators *Â* and *B[combining circumflex]* has nothing to do with the ability to measure them together (see Table 2). This seeming contradiction with the laws of quantum mechanics arises purely from a hardware restriction that one can measure a single qubit at a time. On the other hand, qubit-wise commutativity is still a sufficient but not necessary condition for single-qubit measurability. Removing the single-qubit measurement restriction in the near future will not make our scheme obsolete but rather would allow us to skip the single-particle level. For example, if two-qubit measurements will be available, one can look for two-qubit operators commuting with the Hamiltonian and integrate out pairs of qubits to define next measurable two-qubit operators.

**Table 2:** Commutativity of two operators and their simultaneous single-qubit measurability (SQM)

*Â*	*B[combining circumflex]*	[*Â*, *B[combining circumflex]*]	SQM of (*Â* + *B[combining circumflex]*)
*ẑ*_1_ *ẑ*_2_	*ẑ*_2_ *ẑ*_3_	0	Yes
*ẑ*_1_ *ẑ*_2_	*x[combining circumflex]*_1_ *x[combining circumflex]*_2_	0	No
*ẑ*_1_ *ẑ*_3_	*x[combining circumflex]*_1_ *ẑ*_2_	≠0	Yes
*ẑ*_1_ *ẑ*_2_	*x[combining circumflex]*_1_ *ŷ*_2_	≠0	No

The current approach can address difficulties arising in the exploration of the excited state *via* minimization of variance26
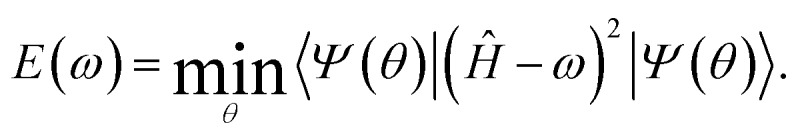



One of the largest practical difficulties is in an increasing number of terms that are required to be measured in eqn (26). Combining some of these terms using the current methodology can reduce the number of needed measurements.

A similar problem with a growing number of terms arises if one would like to obtain the true quantum uncertainty of the measurements for a partitioned Hamiltonian, it requires measuring all covariances between all parts. Ignoring covariances by assuming measurement independence can lead to incorrect estimation of the true uncertainty, both under- and over-estimation are possible.

From the hardware standpoint, the new scheme requires modification of the single-qubit measurement protocol, where measurement results for some qubits will define unitary rotations of other qubits before their measurement, so-called feedforward measurement. This type of measurement has already been implemented in quantum computers based on superconducting^27^ and photonic^19^,^28^,^29^ qubit architectures in the context of measurement-based quantum computing.^15^,^16^ Thus we hope that the new method will become the method of choice for quantum chemistry on a quantum computer in the near future.

## Appendix A: factorization conditions for the Hamiltonian eigenstates

Here we prove that the condition given in the main text for a *N*-qubit Hamiltonian to be in the MF class is actually a necessary and sufficient condition, and hence is a criterion. We will split the proof into two parts: (1) If the Hamiltonian has *N* one-particle operators satisfying the reduction chain, its eigenfunctions can be written as products (sufficiency); (2) if all the Hamiltonian eigenfunctions are in a product form then it will have *N* commuting one-particle operators defined by the reduction scheme (necessity).

(1) Proof of sufficiency: if there exist *N* one-particle operators commuting with a set of reduced Hamiltonians it is straightforward to check that a product of eigenstates of these operators is an eigenstate of the Hamiltonian. Note that any nontrivial one-qubit operator has a non-degenerate spectrum, therefore, there is no degree of freedom related to rotation within a degenerate subspace. The choice of the first eigenstate of the first operator (*Ô*_1_) can define the form of next one-particle operators and their eigenstates.

(2) Proof of necessity: for the *N*-particle eigenstate *Ψ*(1,…*N*) to have a product form it is necessary for the Hamiltonian to have eigenstates of the *φ*_1_(1)*Φ*(2,…*N*) form, where *φ*_1_(1) and *Φ*(2,…*N*) are some arbitrary functions from Hilbert spaces of qubit 1 and *N* – 1 qubits. The latter form is an eigenstate of an operator of the form *Ô*_1_ ⊗ *I*_*N*–1_, where *I*_*N*–1_ is an identity operator and *Ô*_1_ is an operator for which *φ*_1_(1) is an eigenfunction. Then, if the Hamiltonian and *Ô*_1_ ⊗ *I*_*N*–1_ share the eigenstates they must commute. This commutation is equivalent to [*Ĥ*, *Ô*_1_] = 0. The same logic can be applied to *Φ*(2,…*N*) because the next necessary condition for the total eigenfunction of the Hamiltonian to be in a product form is that *Φ*(2,…*N*) = *φ*_2_(2)*Φ̃*(3,…*N*), this gives rise to another commuting operator *Ô*_2_ whose eigenfunction is *φ*_2_. It is important to note though that *Ô*_2_ does not need to commute with *Ĥ* but only with its reduced version *H*_*N*–1_ = = 〈*φ*_1_|*Ĥ*|*φ*_1_〉. This chain can be continued until we reach the end of the variable list.. This chain can be continued until we reach the end of the variable list.

### Many-particle commuting operator extension

Similarly if we can find an *M*-particle operator *Ô* commuting with *Ĥ* then, because of the theorem on commuting operators, there is a common set of eigenfunctions. With multi-qubit operators one needs to be careful because they can have a degenerate spectrum. In the case of the non-degenerate spectrum of *Ô* the common eigenstates have the factorized form *Ψ*(1,…*N*) = *Φ*(1,…*M*)*χ*(*M* + 1,…*N*), which serves as a solid ground for the discussion in the main text. In the degenerate case, the most general form of a common eigenstate is 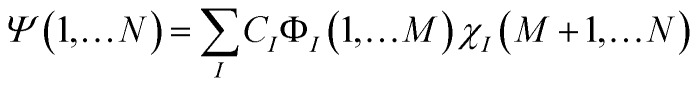, where *ÔΦ*_*I*_(1,…M) = *λΦ*_*I*_(1,…*M*), *I* = 1,…*k*. In this case, the important question becomes whether the Hamiltonian allows for the eigenstates to be single product states,
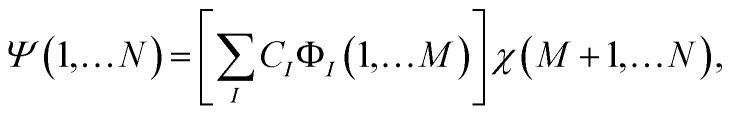
or not? To answer this question one needs to construct a reduced matrix operator within the degenerate subspace {*Φ*_*I*_(1,…*M*)}

27 *Ĥ*_*IJ*_^(*N*–*M*)^ = = 〈*Φ*_*I*_|*Ĥ*|*Φ*_*J*_〉,,

where integration is done over the first *M* variables. If there exists *Φ*_*I*_ for which *Ĥ*_*IJ*_^(*N*–*M*)^ = 0 where *J* ≠ *I* then *Ψ*(1,…*N*) = *Φ*(1,…*M*)*χ*(*M* + 1,…*N*) will be an eigenfunction of the Hamiltonian. For all *Φ*_*I*_(1,…*M*) eigenfunctions to form product states, all off-diagonal elements of *Ĥ*_*IJ*_^(*N*–*M*)^ must be zero. There is one more possibility for the factorized eigenstates, if the reduced matrix operator has the particular form

28 *Ĥ*_*IJ*_^(*N*–*M*)^ = *h*_*IJ*_*Ĥ*^(*N*–*M*)^,

where *h*_*IJ*_ are elements of a constant matrix and *Ĥ*^(*N*–*M*)^ is a single reduced operator acting on *N* – *M* variables. Note that for doing this analysis one needs to be able to obtain only eigenstates of *Ô*. This is presumably an easier procedure since *M* < *N*.

Thus, in the degenerate case, having a product form is not guaranteed and therefore, one may be able to obtain the unitary transformation unentangling qubits only in the described two cases. Yet, finding the commuting operator *Ô* is a necessary condition for the existence of an unentangling unitary transformation.

## Appendix B: illustration of the mean-field partitioning procedure

To illustrate the MF partitioning procedure with a nontrivial example let us consider the model Hamiltonian whose partitioning gives rise to the scheme in Fig. 3

29 *Ĥ* = 3*x[combining circumflex]*_1_*x[combining circumflex]*_2_*x[combining circumflex]*_3_ + *x[combining circumflex]*_1_*x[combining circumflex]*_2_*ŷ*_3_ + 5*x[combining circumflex]*_1_*x[combining circumflex]*_2_*ẑ*_3_ + 5*x[combining circumflex]*_1_*ŷ*_2_*x[combining circumflex]*_3_ + 7*x[combining circumflex]*_1_*ŷ*_2_*ẑ*_3_ + 3*x[combining circumflex]*_1_*ẑ*_2_*x[combining circumflex]*_3_ + *x[combining circumflex]*_1_*ẑ*_2_*ŷ*_3_ + 5*x[combining circumflex]*_1_*ẑ*_2_*ẑ*_3_ + 6*ŷ*_1_*x[combining circumflex]*_2_*x[combining circumflex]*_3_ + 2*ŷ*_1_*x[combining circumflex]*_2_*ŷ*_3_ + 10*ŷ*_1_*x[combining circumflex]*_2_*ẑ*_3_ + 10*ŷ*_1_*ŷ*_2_*x[combining circumflex]*_3_ + 14*ŷ*_1_*ŷ*_2_*ẑ*_3_ + 6*ŷ*_1_*ẑ*_2_*x[combining circumflex]*_3_ + 2*ŷ*_1_*ẑ*_2_*ŷ*_3_ + 10*ŷ*_1_*ẑ*_2_*ẑ*_3_ + 3*ẑ*_1_*x[combining circumflex]*_2_*x[combining circumflex]*_3_ + *ẑ*_1_*x[combining circumflex]*_2_*ŷ*_3_ + 5*ẑ*_1_*x[combining circumflex]*_2_*ẑ*_3_ + 5*ẑ*_1_*ŷ*_2_*x[combining circumflex]*_3_ + 7*ẑ*_1_*ŷ*_2_*ẑ*_3_ + 3*ẑ*_1_*ẑ*_2_*x[combining circumflex]*_3_ + *ẑ*_1_*ẑ*_2_*ŷ*_3_ + 5*ẑ*_1_*ẑ*_2_*ẑ*_3_

To assess whether the partitioning of *Ĥ* is possible based on qubit *k* = 1 we rewrite the Hamiltonian as

30 *Ĥ* = *x[combining circumflex]*_1_*ĥ*_*x*_ + *ŷ*_1_*ĥ*_*y*_ + *ẑ*_1_*ĥ_z_*,

where

31 *ĥ*_*x*_ = 3*x[combining circumflex]*_2_*x[combining circumflex]*_3_ + *x[combining circumflex]*_2_*ŷ*_3_ + 5*x[combining circumflex]*_2_*ẑ*_3_ + 5*ŷ*_2_*x[combining circumflex]*_3_ + 7*ŷ*_2_*ẑ*_3_ + 3*ẑ*_2_*x[combining circumflex]*_3_ + *ẑ*_2_*ŷ*_3_ + 5*ẑ*_2_*ẑ*_3_

32 *ĥ*_*y*_ = 6*x[combining circumflex]*_2_*x[combining circumflex]*_3_ + 2*x[combining circumflex]*_2_*ŷ*_3_ + 10*x[combining circumflex]*_2_*ẑ*_3_ + 10*ŷ*_2_*x[combining circumflex]*_3_ + 14*ŷ*_2_*ẑ*_3_ + 6*ẑ*_2_*x[combining circumflex]*_3_ + 2*ẑ*_2_*ŷ*_3_ + 10*ẑ*_2_*ẑ*_3_

33 *ĥ*_*z*_ = 3*x[combining circumflex]*_2_*x[combining circumflex]*_3_ + *x[combining circumflex]*_2_*ŷ*_3_ + 5*x[combining circumflex]*_2_*ẑ*_3_ + 5*ŷ*_2_*x[combining circumflex]*_3_ + 7*ŷ*_2_*ẑ*_3_ + 3*ẑ*_2_*x[combining circumflex]*_3_ + *ẑ*_2_*ŷ*_3_ + 5*ẑ*_2_*ẑ*_3_

Each *ĥ*_*x*,*y*,*z*_ is transformed into a vector. For example34
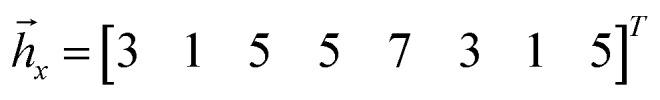
in the basis {*x[combining circumflex]*_2_*x[combining circumflex]*_3_, *x[combining circumflex]*_2_*ŷ*_3_, *x[combining circumflex]*_2_*ẑ*_3_, *ŷ*_2_*x[combining circumflex]*_3_, *ŷ*_2_*ẑ*_3_, *ẑ*_2_*x[combining circumflex]*_3_, *ẑ*_2_*ŷ*_3_, *ẑ*_2_*ẑ*_3_}. *S*_1_ is obtained as *A*†1*A*_1_, where 
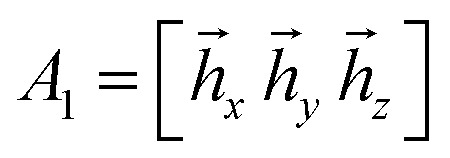
. Diagonalizing of *S*_1_ gives one non-zero eigenvalue *d* and a corresponding eigenvector 
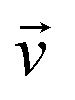
. The dimensionality of the *S*_1_ kernel is 2, *l*(1) = 2, and it implies collinearity of 
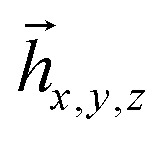
 (Fig. 2a). Performing similar analysis for *S*_2_ and *S*_3_, one can find *l*(2) = *l*(3) = 1 (see Fig. 3). Therefore, we rewrite the Hamiltonian as *Ĥ* = *ĥ*(2,3)*Ô*_1_, where

35 *Ô*_1_ = 0.408248*x[combining circumflex]*_1_ + 0.816497*ŷ*_1_ + 0.408248*ẑ*_1_

36 *ĥ*(2,3) = 7.34847*x[combining circumflex]*_2_*x[combining circumflex]*_3_ + 2.44949*x[combining circumflex]*_2_*ŷ*_3_ + 12.2474*x[combining circumflex]*_2_*ẑ*_3_ + 12.2474*ŷ*_2_*x[combining circumflex]*_3_ + 17.1464*ŷ*_2_*ẑ*_3_ + 7.34847*ẑ*_2_*x[combining circumflex]*_3_ + 2.44949*ẑ*_2_*ŷ*_3_ + 12.2474*ẑ*_2_*ẑ*_3_

*Ô*_1_ and *ĥ*(2,3) were obtained through a linear combination of {*x[combining circumflex]*_1_, *ŷ*_1_, *ẑ*_1_} and {*ĥ*_*x*_, *ĥ_y_*, *ĥ_z_*} with coefficients from the eigenvector 
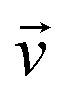
.

As the next step, we consider *ĥ*(2,3), it can be partitioned based on either qubit *k* = 2 or *k* = 3. Both qubits have the same values of *l*(*k*) = 1 and 
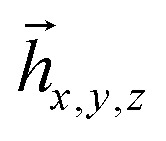
 are in a single plane (Fig. 2b). Here, we choose arbitrarily *k* = 2, diagonalizing *S*_2_ leads to two non-zero eigenvalues (*d*_1_,*d*_2_) and corresponding eigenvectors 
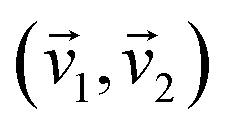
. Following the procedure, *ĥ*(2,3) decomposes to37
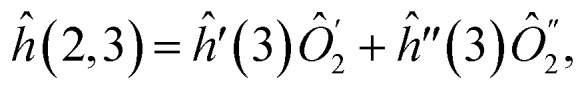
where38
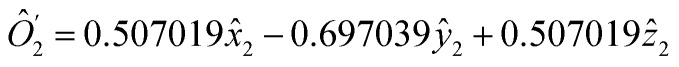

39 *ĥ*′(3)=–1.08532*x[combining circumflex]*_3_ + 2.48388*ŷ*_3_ + 0.467647*ẑ*_3_

40
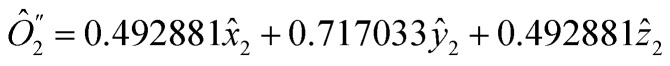

41 *ĥ*′′(3) = 16.0257*x[combining circumflex]*_3_ + 2.41461*ŷ*_3_ + 24.3676*ẑ*_3_.

The single-qubit operators 
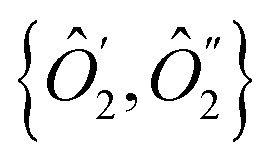
 and their complements {*ĥ*′, *ĥ*′′} were obtained taking linear combinations of {*x[combining circumflex]*_2_, *ŷ*_2_, *ẑ*_2_} and {*ĥ*_*x*_, *ĥ*_*y*_, *ĥ_z_*} with coefficients from the eigenvectors 
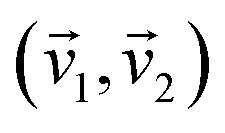
, respectively.

The complexity of a single step of the MF partitioning procedure is polynomial with the number of qubits. In each step we need to evaluate the *l*(*k*) function for each of the qubits present. Evaluation of the *l*(*k*) function requires building the corresponding overlap matrix *S*_*k*_, which involves inner products between columns of *A*_*k*_ matrices. Since the length of *A*_*k*_ columns (*h[combining macron]*_*x*,*y*,*z*_) scales as *N*^4^ at most (this is the scaling of the total number of terms in the Hamiltonian), the construction of *S*_*k*_ scales as *N*^4^ as well. Thus funding *l*(*k*) functions for all qubits in general has *O*(*N*^5^) scaling.

## Appendix C: Hamiltonian details

### H_2_ molecule

One- and two-electron integrals in the canonical restricted Hartree–Fock (RHF) molecular orbital basis for *R*(H–H) = 1.5 Å were used in the BK transformation to produce the corresponding qubit Hamiltonian. Spin-orbitals were alternating in the order *α*, *β*, *α*, …. The explicit expression for the BK qubit Hamiltonian is given in the ESI.†

### LiH molecule

A qubit Hamiltonian for *R*(Li–H) = 3.2 Å distance was generated using the parity fermion-to-qubit transformation.^30^ Spin-orbitals were arranged as “first all alpha then all beta” in the fermionic form; since there are 3 active molecular orbitals in the problem, this leads to a 6-qubit Hamiltonian. Further details on the Hamiltonian are given in the ESI.†

## Conflicts of interest

There are no conflicts of interest to declare.

## Supplementary Material

Supplementary information
