# Supplementary material for: Revising the measurement process in the variational quantum eigensolver: is it possible to reduce the number of separately measured operators?
Source: Chem Sci. 2019 Feb 12;10(13):3746–55. doi: 10.1039/c8sc05592k (PMC6461116; doi:10.1039/c8sc05592k)
Supplement: Supplementary file 1 [file SC-010-C8SC05592K-s001.pdf]

Electronic Supplementary Material (ESI) for Chemical Science.
This journal is © The Royal Society of Chemistry 2019

Supplementary material for "Revising measurement process in the variational quantum eigensolver: Is it possible to reduce the number of separately measured operators?"

November 7, 2018

1 Hamiltonians for H_2, R(H-H)=1.5Å

The BK transformed qubit Hamiltonian is

$$\begin{aligned}\hat{H}_{\mathrm{H}_2} \quad = \quad & -0.491786 + 0.0934565 z_4 + 0.138176 z_3 + 0.0934565 z_3 z_4 + 0.057384 x_2 z_3 x_4 \\ & +0.057384 y_2 z_3 y_4 - 0.0356448 z_2 + 0.0825371 z_2 z_4 + 0.139921 z_2 z_3 z_4 + 0.145855 z_1 z_3 \\ & +0.057384 z_1 x_2 z_3 x_4 + 0.057384 z_1 y_2 z_3 y_4 + 0.0825371 z_1 z_2 z_4 - 0.0356448 z_1 z_2 z_3 \\ & +0.139921 z_1 z_2 z_3 z_4. \qquad (1)\end{aligned}$$

After reduction of the 1st and 3rd qubits, the reduced Hamiltonian is

$$\hat{H}_{24} = -0.207755 + 0.186913 z_4 + 0.114768 x_2 x_4 + 0.114768 y_2 y_4 - 0.0712896 z_2 + 0.444916 z_2 z_4 \quad (2)$$

There are 3 QWC or MF terms in $\hat{H}_{24}$.

$$\hat{H}_{24}^{(1)} \quad = \quad -0.207755 + 0.186913 z_4 - 0.0712896 z_2 + 0.444916 z_2 z_4 \quad (3)$$
$$\hat{H}_{24}^{(2)} \quad = \quad 0.114768 x_2 x_4 \quad (4)$$
$$\hat{H}_{24}^{(3)} \quad = \quad 0.114768 y_2 y_4. \quad (5)$$

2 Hamiltonians for LiH R(Li-H)=3.2 Å

Without reduction, the original BK transformed Hamiltonian is

$$
\begin{aligned}
\hat{H}^{(6)}_{LiH} \quad = \quad & -7.25623 + 0.0193211x_1 - 0.0550145z_1 - 0.0206781x_2 - 0.0117773x_2x_1 - 0.00168681x_2z_1 \\
& -0.0248725y_2y_1 + 0.040105z_2 - 0.0193211z_2x_1 - 0.11089z_2z_1 + 0.00262206z_3x_1 + 0.0477866z_3z_1 \\
& +0.00168681z_3x_2 - 0.0248725z_3x_2x_1 + 0.0206781z_3x_2z_1 - 0.0117773z_3y_2y_1 - 0.16267z_3z_2 \\
& +0.0536044z_3z_2z_1 + 0.0193211x_4 + 0.0195265x_4x_1 + 0.024665x_4z_1 - 0.0152095x_4x_2 \\
& -0.0195265x_4z_2x_1 - 0.00184858x_4z_2z_1 - 0.019804x_4z_3x_2x_1 + 0.0152095x_4z_3x_2z_1 \\
& +0.00981459x_4z_3z_2 - 0.0211245z_4x_2x_1 + 0.0214908z_4x_2z_1 + 0.0815464z_4z_2 - 0.0550145z_4z_3 \\
& +0.024665z_4z_3x_1 + 0.0977784z_4z_3z_1 - 0.0214908z_4z_3x_2 - 0.0211245z_4z_3y_2y_1 \\
& +0.0596316z_4z_3z_2z_1 - 0.0206781x_5 - 0.0152095x_5x_1 - 0.0214908x_5z_1 + 0.0165101x_5x_2 \\
& +0.0152095x_5z_2x_1 + 0.00607138x_5z_2z_1 + 0.0124366x_5z_3x_2x_1 - 0.0165101x_5z_3x_2z_1 \\
& -0.0117773x_5x_4 - 0.00168681x_5z_4z_3 - 0.0248725y_5y_4 - 0.019804y_5y_4x_1 - 0.0211245y_5y_4z_1 \\
& +0.0124366y_5y_4x_2 + 0.0279419y_5y_4y_2y_1 + 0.019804y_5y_4z_2x_1 - 0.00343219y_5y_4z_2z_1 \\
& +0.0279419y_5y_4z_3x_2x_1 - 0.0124366y_5y_4z_3x_2z_1 - 0.00459719y_5y_4z_3z_2 + 0.040105z_5z_3 \\
& +0.019804z_5x_4x_2x_1 - 0.0152095z_5x_4x_2z_1 - 0.00981459z_5x_4z_2 - 0.0193211z_5x_4z_3 \\
& -0.024665z_5x_4z_3z_1 + 0.0152095z_5x_4z_3x_2 + 0.019804z_5x_4z_3y_2y_1 + 0.0195265z_5x_4z_3z_2x_1 \\
& +0.00184858z_5x_4z_3z_2z_1 - 0.11089z_5z_4 - 0.00184858z_5z_4x_1 + 0.0596316z_5z_4z_1 \\
& +0.00607138z_5z_4x_2 - 0.00343219z_5z_4y_2y_1 + 0.00184858z_5z_4z_2x_1 + 0.0717285z_5z_4z_2z_1 \\
& -0.00343219z_5z_4z_3x_2x_1 - 0.00607138z_5z_4z_3x_2z_1 + 0.0642967z_5z_4z_3z_2 + 0.00262206z_6x_4z_3 \\
& +0.0477866z_6z_4 + 0.00168681z_6x_5z_3 + 0.0279419z_6x_5x_4x_2x_1 - 0.0124366z_6x_5x_4x_2z_1 \\
& -0.00459719z_6x_5x_4z_2 - 0.0248725z_6x_5x_4z_3 - 0.019804z_6x_5x_4z_3x_1 - 0.0211245z_6x_5x_4z_3z_1 \\
& +0.0124366z_6x_5x_4z_3x_2 + 0.0279419z_6x_5x_4z_3y_2y_1 + 0.019804z_6x_5x_4z_3z_2x_1 \\
& -0.00343219z_6x_5x_4z_3z_2z_1 + 0.0206781z_6x_5z_4 + 0.0152095z_6x_5z_4x_1 + 0.0214908z_6x_5z_4z_1 \\
& -0.0165101z_6x_5z_4x_2 - 0.0124366z_6x_5z_4y_2y_1 - 0.0152095z_6x_5z_4z_2x_1 - 0.00607138z_6x_5z_4z_2z_1 \\
& -0.0124366z_6x_5z_4z_3x_2x_1 + 0.0165101z_6x_5z_4z_3x_2z_1 + 0.00857088z_6x_5z_4z_3z_2 \\
& -0.16267z_6z_5 + 0.00981459z_6z_5x_1 + 0.0815464z_6z_5z_1 - 0.00857088z_6z_5x_2 - 0.00459719z_6z_5y_2y_1 \\
& -0.00981459z_6z_5z_2x_1 + 0.0642967z_6z_5z_2z_1 - 0.00459719z_6z_5z_3x_2x_1 - 0.00262206z_3z_2x_1 \\
& +0.00857088z_6z_5z_3x_2z_1 + 0.0800052z_6z_5z_3z_2 - 0.00262206z_6z_5x_4 + 0.0536044z_6z_5z_4z_3 \\
& -0.019804x_4y_2y_1 + 0.0124366x_5y_2y_1 - 0.024665z_4z_3z_2x_1 - 0.0117773z_6y_5y_4z_3 \\
& -0.0195265z_5x_4z_3x_1 - 0.00857088x_5z_3z_2 \qquad (6)
\end{aligned}
$$

After reducing the 3rd and 6th qubits, the LiH Hamiltonian becomes

$$
\begin{aligned}
\hat{H}_{LiH} \quad = \quad & -7.25623 + 0.016699 x_1 - 0.102801 z_1 - 0.0223649 x_2 + 0.0130951 x_2 x_1 - 0.0223649 x_2 z_1 \\
& -0.0130951 y_2 y_1 + 0.202775 z_2 - 0.016699 z_2 x_1 - 0.164495 z_2 z_1 + 0.016699 x_3 \\
& +0.0195265 x_3 x_1 + 0.024665 x_3 z_1 - 0.0152095 x_3 x_2 + 0.019804 x_3 x_2 x_1 \\
& -0.0152095 x_3 x_2 z_1 - 0.019804 x_3 y_2 y_1 - 0.00981459 x_3 z_2 - 0.0195265 x_3 z_2 x_1 \\
& -0.00184858 x_3 z_2 z_1 + 0.102801 z_3 - 0.024665 z_3 x_1 - 0.0977784 z_3 z_1 + 0.0214908 z_3 x_2 \\
& -0.0211245 z_3 x_2 x_1 + 0.0214908 z_3 x_2 z_1 + 0.0211245 z_3 y_2 y_1 + 0.0815464 z_3 z_2 \\
& +0.024665 z_3 z_2 x_1 - 0.0596316 z_3 z_2 z_1 - 0.0223649 x_4 - 0.0152095 x_4 x_1 - 0.0214908 x_4 z_1 \\
& +0.0165101 x_4 x_2 - 0.0124366 x_4 x_2 x_1 + 0.0165101 x_4 x_2 z_1 + 0.0124366 x_4 y_2 y_1 \\
& +0.00857088 x_4 z_2 + 0.0152095 x_4 z_2 x_1 + 0.00607138 x_4 z_2 z_1 + 0.0130951 x_4 x_3 \\
& +0.019804 x_4 x_3 x_1 + 0.0211245 x_4 x_3 z_1 - 0.0124366 x_4 x_3 x_2 + 0.0279419 x_4 x_3 x_2 x_1 \\
& -0.0124366 x_4 x_3 x_2 z_1 - 0.0279419 x_4 x_3 y_2 y_1 - 0.00459719 x_4 x_3 z_2 - 0.019804 x_4 x_3 z_2 x_1 \\
& +0.00343219 x_4 x_3 z_2 z_1 + 0.0223649 x_4 z_3 + 0.0152095 x_4 z_3 x_1 + 0.0214908 x_4 z_3 z_1 \\
& -0.0165101 x_4 z_3 x_2 + 0.0124366 x_4 z_3 x_2 x_1 - 0.0165101 x_4 z_3 x_2 z_1 - 0.0124366 x_4 z_3 y_2 y_1 \\
& -0.00857088 x_4 z_3 z_2 - 0.0152095 x_4 z_3 z_2 x_1 - 0.00607138 x_4 z_3 z_2 z_1 - 0.0130951 y_4 y_3 \\
& -0.019804 y_4 y_3 x_1 - 0.0211245 y_4 y_3 z_1 + 0.0124366 y_4 y_3 x_2 - 0.0279419 y_4 y_3 x_2 x_1 \\
& +0.0124366 y_4 y_3 x_2 z_1 + 0.0279419 y_4 y_3 y_2 y_1 + 0.00459719 y_4 y_3 z_2 + 0.019804 y_4 y_3 z_2 x_1 \\
& -0.00343219 y_4 y_3 z_2 z_1 - 0.202775 z_4 + 0.00981459 z_4 x_1 + 0.0815464 z_4 z_1 - 0.00857088 z_4 x_2 \\
& +0.00459719 z_4 x_2 x_1 - 0.00857088 z_4 x_2 z_1 - 0.00459719 z_4 y_2 y_1 - 0.0800052 z_4 z_2 \\
& -0.00981459 z_4 z_2 x_1 + 0.0642967 z_4 z_2 z_1 + 0.016699 z_4 x_3 + 0.0195265 z_4 x_3 x_1 \\
& +0.024665 z_4 x_3 z_1 - 0.0152095 z_4 x_3 x_2 + 0.019804 z_4 x_3 x_2 x_1 - 0.0152095 z_4 x_3 x_2 z_1 \\
& -0.019804 z_4 x_3 y_2 y_1 - 0.00981459 z_4 x_3 z_2 - 0.0195265 z_4 x_3 z_2 x_1 - 0.00184858 z_4 x_3 z_2 z_1 \\
& -0.164495 z_4 z_3 - 0.00184858 z_4 z_3 x_1 + 0.0596316 z_4 z_3 z_1 + 0.00607138 z_4 z_3 x_2 \\
& +0.00343219 z_4 z_3 x_2 x_1 + 0.00607138 z_4 z_3 x_2 z_1 - 0.00343219 z_4 z_3 y_2 y_1 \\
& -0.0642967 z_4 z_3 z_2 + 0.00184858 z_4 z_3 z_2 x_1 + 0.0717285 z_4 z_3 z_2 z_1 \qquad (7)
\end{aligned}
$$

$\hat{H}_{LiH}$ contains 25 QWC terms

$$h_1 = 0.0279419x_4x_3x_2x_1 \quad (8)$$
$$h_2 = 0.019804x_3x_2x_1 + 0.019804z_4x_3x_2x_1 \quad (9)$$
$$h_3 = -0.0279419y_4y_3x_2x_1 \quad (10)$$
$$h_4 = -0.0124366x_4x_2x_1 + 0.0124366x_4z_3x_2x_1 \quad (11)$$
$$h_5 = 0.0130951x_2x_1 - 0.0211245z_3x_2x_1 + 0.00459719z_4x_2x_1 + 0.00343219z_4z_3x_2x_1 \quad (12)$$
$$h_6 = 0.019804x_4x_3x_1 - 0.019804x_4x_3z_2x_1 \quad (13)$$
$$h_7 = 0.0195265x_3x_1 - 0.0195265x_3z_2x_1 + 0.0195265z_4x_3x_1 - 0.0195265z_4x_3z_2x_1 \quad (14)$$
$$h_8 = -0.019804y_4y_3x_1 + 0.019804y_4y_3z_2x_1 \quad (15)$$
$$h_9 = -0.0152095x_4x_1 + 0.0152095x_4z_2x_1 + 0.0152095x_4z_3x_1 - 0.0152095x_4z_3z_2x_1 \quad (16)$$
$$h_{10} = 0.016699x_1 - 0.016699z_2x_1 - 0.024665z_3x_1 + 0.024665z_3z_2x_1 + 0.00981459z_4x_1$$
$$-0.00981459z_4z_2x_1 - 0.00184858z_4z_3x_1 + 0.00184858z_4z_3z_2x_1 \quad (17)$$
$$h_{11} = -0.0279419x_4x_3y_2y_1 \quad (18)$$
$$h_{12} = -0.019804x_3y_2y_1 - 0.019804z_4x_3y_2y_1 \quad (19)$$
$$h_{13} = 0.0279419y_4y_3y_2y_1 \quad (20)$$
$$h_{14} = 0.0124366x_4y_2y_1 - 0.0124366x_4z_3y_2y_1 \quad (21)$$
$$h_{15} = -0.0130951y_2y_1 + 0.0211245z_3y_2y_1 - 0.00459719z_4y_2y_1 - 0.00343219z_4z_3y_2y_1 \quad (22)$$
$$h_{16} = -0.0124366x_4x_3x_2 - 0.0124366x_4x_3x_2z_1 \quad (23)$$
$$h_{17} = -0.0152095x_3x_2 - 0.0152095x_3x_2z_1 - 0.0152095z_4x_3x_2 - 0.0152095z_4x_3x_2z_1 \quad (24)$$
$$h_{18} = 0.0124366y_4y_3x_2 + 0.0124366y_4y_3x_2z_1 \quad (25)$$
$$h_{19} = 0.0165101x_4x_2 + 0.0165101x_4x_2z_1 - 0.0165101x_4z_3x_2 - 0.0165101x_4z_3x_2z_1 \quad (26)$$
$$h_{20} = -0.0223649x_2 - 0.0223649x_2z_1 + 0.0214908z_3x_2 + 0.0214908z_3x_2z_1 - 0.00857088z_4x_2 \quad (27)$$
$$-0.00857088z_4x_2z_1 + 0.00607138z_4z_3x_2 + 0.00607138z_4z_3x_2z_1 \quad (28)$$
$$h_{21} = 0.0130951x_4x_3 + 0.0211245x_4x_3z_1 - 0.00459719x_4x_3z_2 + 0.00343219x_4x_3z_2z_1 \quad (29)$$
$$h_{22} = 0.016699x_3 + 0.024665x_3z_1 - 0.00981459x_3z_2 - 0.00184858x_3z_2z_1 + 0.016699z_4x_3$$
$$+0.024665z_4x_3z_1 - 0.00981459z_4x_3z_2 - 0.00184858z_4x_3z_2z_1 \quad (30)$$
$$h_{23} = -0.0130951y_4y_3 - 0.0211245y_4y_3z_1 + 0.00459719y_4y_3z_2 - 0.00343219y_4y_3z_2z_1 \quad (31)$$
$$h_{24} = -0.0223649x_4 - 0.0214908x_4z_1 + 0.00857088x_4z_2 + 0.00607138x_4z_2z_1 + 0.0223649x_4z_3$$
$$+0.0214908x_4z_3z_1 - 0.00857088x_4z_3z_2 - 0.00607138x_4z_3z_2z_1 \quad (32)$$
$$h_{25} = -7.25623 - 0.102801z_1 + 0.202775z_2 - 0.164495z_2z_1 + 0.102801z_3 - 0.0977784z_3z_1 + 0.0815464z_3z_2$$
$$-0.0596316z_3z_2z_1 - 0.202775z_4 + 0.0815464z_4z_1 - 0.0800052z_4z_2 + 0.0642967z_4z_2z_1$$
$$-0.164495z_4z_3 + 0.0596316z_4z_3z_1 - 0.0642967z_4z_3z_2 + 0.0717285z_4z_3z_2z_1. \quad (33)$$

$\hat{H}_{LiH}$ can be split into 13 MF terms (MF-partitioning 1p in Table I of the

main text)

$$
\begin{aligned}
\tilde{h}_1 &= 0.0124366 y_4 y_3 x_2 - 0.0279419 y_4 y_3 x_2 x_1 + 0.0124366 y_4 y_3 x_2 z_1 && (34)\\
\tilde{h}_2 &= -0.019804 x_3 y_2 y_1 - 0.0279419 x_4 x_3 y_2 y_1 - 0.019804 z_4 x_3 y_2 y_1 && (35)\\
\tilde{h}_3 &= 0.0279419 y_4 y_3 y_2 y_1 && (36)\\
\tilde{h}_4 &= -0.0130951 y_2 y_1 + 0.0211245 z_3 y_2 y_1 + 0.0124366 x_4 y_2 y_1 \\
&\quad -0.0124366 x_4 z_3 y_2 y_1 - 0.00459719 z_4 y_2 y_1 - 0.00343219 z_4 z_3 y_2 y_1 && (37)\\
\tilde{h}_5 &= -0.0130951 y_4 y_3 - 0.019804 y_4 y_3 x_1 - 0.0211245 y_4 y_3 z_1 \\
&\quad +0.00459719 y_4 y_3 z_2 + 0.019804 y_4 y_3 z_2 x_1 - 0.00343219 y_4 y_3 z_2 z_1 && (38)\\
\tilde{h}_6 &= 0.019804 x_3 x_2 x_1 + 0.0279419 x_4 x_3 x_2 x_1 + 0.019804 z_4 x_3 x_2 x_1 && (39)\\
\tilde{h}_7 &= -0.0152095 x_3 x_2 - 0.0152095 x_3 x_2 z_1 - 0.0124366 x_4 x_3 x_2 \\
&\quad -0.0124366 x_4 x_3 x_2 z_1 - 0.0152095 z_4 x_3 x_2 - 0.0152095 z_4 x_3 x_2 z_1 && (40)\\
\tilde{h}_8 &= 0.0130951 x_2 x_1 - 0.0211245 z_3 x_2 x_1 - 0.0124366 x_4 x_2 x_1 \\
&\quad +0.0124366 x_4 z_3 x_2 x_1 + 0.00459719 z_4 x_2 x_1 + 0.00343219 z_4 z_3 x_2 x_1 && (41)\\
\tilde{h}_9 &= -0.0223649 x_2 - 0.0223649 x_2 z_1 + 0.0214908 z_3 x_2 + 0.0214908 z_3 x_2 z_1 + 0.0165101 x_4 x_2 \\
&\quad +0.0165101 x_4 x_2 z_1 - 0.0165101 x_4 z_3 x_2 - 0.0165101 x_4 z_3 x_2 z_1 - 0.00857088 z_4 x_2 \\
&\quad -0.00857088 z_4 x_2 z_1 + 0.00607138 z_4 z_3 x_2 + 0.00607138 z_4 z_3 x_2 z_1 && (42)\\
\tilde{h}_{10} &= 0.0195265 x_3 x_1 - 0.0195265 x_3 z_2 x_1 + 0.019804 x_4 x_3 x_1 \\
&\quad -0.019804 x_4 x_3 z_2 x_1 + 0.0195265 z_4 x_3 x_1 - 0.0195265 z_4 x_3 z_2 x_1 && (43)\\
\tilde{h}_{11} &= 0.016699 x_3 + 0.024665 x_3 z_1 - 0.00981459 x_3 z_2 - 0.00184858 x_3 z_2 z_1 + 0.0130951 x_4 x_3 \\
&\quad +0.0211245 x_4 x_3 z_1 - 0.00459719 x_4 x_3 z_2 + 0.00343219 x_4 x_3 z_2 z_1 + 0.016699 z_4 x_3 \\
&\quad +0.024665 z_4 x_3 z_1 - 0.00981459 z_4 x_3 z_2 - 0.00184858 z_4 x_3 z_2 z_1 && (44)\\
\tilde{h}_{12} &= 0.016699 x_1 - 0.016699 z_2 x_1 - 0.024665 z_3 x_1 + 0.024665 z_3 z_2 x_1 - 0.0152095 x_4 x_1 \\
&\quad +0.0152095 x_4 z_2 x_1 + 0.0152095 x_4 z_3 x_1 - 0.0152095 x_4 z_3 z_2 x_1 + 0.00981459 z_4 x_1 \\
&\quad -0.00981459 z_4 z_2 x_1 - 0.00184858 z_4 z_3 x_1 + 0.00184858 z_4 z_3 z_2 x_1 && (45)\\
\tilde{h}_{13} &= -7.25623 - 0.102801 z_1 + 0.202775 z_2 - 0.164495 z_2 z_1 + 0.102801 z_3 - 0.0977784 z_3 z_1 \\
&\quad +0.0815464 z_3 z_2 - 0.0596316 z_3 z_2 z_1 - 0.0223649 x_4 - 0.0214908 x_4 z_1 \\
&\quad +0.00857088 x_4 z_2 + 0.00607138 x_4 z_2 z_1 + 0.0223649 x_4 z_3 + 0.0214908 x_4 z_3 z_1 \\
&\quad -0.00857088 x_4 z_3 z_2 - 0.00607138 x_4 z_3 z_2 z_1 - 0.202775 z_4 + 0.0815464 z_4 z_1 \\
&\quad -0.0800052 z_4 z_2 + 0.0642967 z_4 z_2 z_1 - 0.164495 z_4 z_3 + 0.0596316 z_4 z_3 z_1 \\
&\quad -0.0642967 z_4 z_3 z_2 + 0.0717285 z_4 z_3 z_2 z_1. && (46)
\end{aligned}
$$

Involving 2-qubit transformations before the single-qubit measurement, $\hat{H}_{LiH}$ can be treated by splitting into the following 5 terms (MF-partitioning 2p in

Table I of the main text)

$$\begin{aligned}
\tilde{H}_1^{(2p)} &= 0.0130951x_2x_1 + 0.019804x_3x_2x_1 - 0.0211245z_3x_2x_1 - 0.0124366x_4x_2x_1 \\
&+0.0279419x_4x_3x_2x_1 + 0.0124366x_4z_3x_2x_1 - 0.0279419y_4y_3x_2x_1 \\
&+0.00459719z_4x_2x_1 + 0.019804z_4x_3x_2x_1 + 0.00343219z_4z_3x_2x_1 \quad (47)
\end{aligned}$$

$$\begin{aligned}
\tilde{H}_2^{(2p)} &= 0.016699x_1 - 0.016699z_2x_1 + 0.0195265x_3x_1 - 0.0195265x_3z_2x_1 - 0.024665z_3x_1 \\
&+0.024665z_3z_2x_1 - 0.0152095x_4x_1 + 0.0152095x_4z_2x_1 + 0.019804x_4x_3x_1 \\
&-0.019804x_4x_3z_2x_1 + 0.0152095x_4z_3x_1 - 0.0152095x_4z_3z_2x_1 - 0.019804y_4y_3x_1 \\
&+0.019804y_4y_3z_2x_1 + 0.00981459z_4x_1 - 0.00981459z_4z_2x_1 + 0.0195265z_4x_3x_1 \\
&-0.0195265z_4x_3z_2x_1 - 0.00184858z_4z_3x_1 + 0.00184858z_4z_3z_2x_1 \quad (48)
\end{aligned}$$

$$\begin{aligned}
\tilde{H}_3^{(2p)} &= -0.0130951y_2y_1 - 0.019804x_3y_2y_1 + 0.0211245z_3y_2y_1 + 0.0124366x_4y_2y_1 \\
&-0.0279419x_4x_3y_2y_1 - 0.0124366x_4z_3y_2y_1 + 0.0279419y_4y_3y_2y_1 \\
&-0.00459719z_4y_2y_1 - 0.019804z_4x_3y_2y_1 - 0.00343219z_4z_3y_2y_1 \quad (49)
\end{aligned}$$

$$\begin{aligned}
\tilde{H}_4^{(2p)} &= -0.0223649x_2 - 0.0223649x_2z_1 - 0.0152095x_3x_2 - 0.0152095x_3x_2z_1 + 0.0214908z_3x_2 \\
&+0.0214908z_3x_2z_1 + 0.0165101x_4x_2 + 0.0165101x_4x_2z_1 - 0.0124366x_4x_3x_2 \\
&-0.0124366x_4x_3x_2z_1 - 0.0165101x_4z_3x_2 - 0.0165101x_4z_3x_2z_1 + 0.0124366y_4y_3x_2 \\
&+0.0124366y_4y_3x_2z_1 - 0.00857088z_4x_2 - 0.00857088z_4x_2z_1 - 0.0152095z_4x_3x_2 \\
&-0.0152095z_4x_3x_2z_1 + 0.00607138z_4z_3x_2 + 0.00607138z_4z_3x_2z_1 \quad (50)
\end{aligned}$$

$$\begin{aligned}
\tilde{H}_5^{(2p)} &= -7.25623 - 0.102801z_1 + 0.202775z_2 - 0.164495z_2z_1 + 0.016699x_3 + 0.024665x_3z_1 \\
&-0.00981459x_3z_2 - 0.00184858x_3z_2z_1 + 0.102801z_3 - 0.0977784z_3z_1 + 0.0815464z_3z_2 \\
&-0.0596316z_3z_2z_1 - 0.0223649x_4 - 0.0214908x_4z_1 + 0.00857088x_4z_2 \\
&+0.00607138x_4z_2z_1 + 0.0130951x_4x_3 + 0.0211245x_4x_3z_1 - 0.00459719x_4x_3z_2 \\
&+0.00343219x_4x_3z_2z_1 + 0.0223649x_4z_3 + 0.0214908x_4z_3z_1 - 0.00857088x_4z_3z_2 \\
&-0.00607138x_4z_3z_2z_1 - 0.0130951y_4y_3 - 0.0211245y_4y_3z_1 + 0.00459719y_4y_3z_2 \\
&-0.00343219y_4y_3z_2z_1 - 0.202775z_4 + 0.0815464z_4z_1 - 0.0800052z_4z_2 \\
&+0.0642967z_4z_2z_1 + 0.016699z_4x_3 + 0.024665z_4x_3z_1 - 0.00981459z_4x_3z_2 \\
&-0.00184858z_4x_3z_2z_1 - 0.164495z_4z_3 + 0.0596316z_4z_3z_1 - 0.0642967z_4z_3z_2 \\
&+0.0717285z_4z_3z_2z_1 \quad (51)
\end{aligned}$$
